# Marine-Derived *Bacillus* Biosurfactants as Potential Antibacterial and Antibiofilm Agents Against Oral Pathogens

**DOI:** 10.3390/microorganisms14030573

**Published:** 2026-03-03

**Authors:** Thangaraj Vaishnavi, Elangovan Elavarashi

**Affiliations:** Medical Probiotics Lab, Department of Biotechnology, Sri Ramachandra Faculty of Biomedical Sciences & Technology, Sri Ramachandra Institute of Higher Education and Research, Chennai 600116, Tamil Nadu, India; vaishnavit@sriramachandra.edu.in

**Keywords:** biosurfactants, marine, *Bacillus* species, antibacterial, antibiofilm, oral pathogens

## Abstract

The growing antimicrobial resistance (AMR) is threatening traditional treatments for oral diseases like dental caries and periodontitis, which constitute a significant global health burden. The study aimed to isolate *Bacillus* species from marine samples, to assess their biosurfactant-producing capabilities, and to evaluate their antibacterial activity against oral pathogens. *Bacillus* strains were isolated from marine water and sediment samples, identified by phenotypic and genotypic methods, and screened for their biosurfactant-producing ability by drop collapse, hemolytic activity, bacterial adhesion to hydrocarbons (BATH), oil displacement, and emulsification assays. Ethyl acetate extracts of these *Bacillus* strains were tested for antibacterial efficacy against four oral pathogens (MTCC strains) by the agar-well diffusion method. Among 81 bacterial isolates, 13 were confirmed as *Bacillus* species by phenotypic and 16S rRNA gene sequencing. Six *Bacillus* isolates displayed significant antibacterial activity, and the majority were beta-hemolytic. *Bacillus* strain TVD12 (50 mg/mL) exhibited superior performance by inhibiting *S. mutans* (31 mm ± 0)*, S. anginosus* (30.5 mm ± 0.7), *S. aureus* (20 mm ± 1.4), and *E. faecalis* (29 mm ± 4.24). *Bacillus* strain TVW12 (500 μg/mL) performs better in antibiofilm activity by inhibiting *E. faecalis* 90%, *S. aureus* 87.4%, and *S. mutans* 76.8%. Statistical analysis revealed a distinct dual-activity profile, characterized by consistent broad-spectrum antimicrobial efficacy (*p* = 0.809) alongside specialized, pathogen-specific antibiofilm inhibition (*p* = 0.004). Marine-derived *Bacillus* strains, such as TVW12, and TVD12 demonstrated effective antibacterial and antibiofilm properties, offering a feasible approach to combat oral pathogens, contributing to sustainable development goals (SDGs) by addressing the challenges of antimicrobial resistance (SDG 3) through sustainable marine bioprospecting (SDG 14). These findings suggest their possibility in developing novel antibacterial agents against oral pathogens in future therapeutic applications.

## 1. Introduction

Oral diseases affect approximately 3.5 billion people worldwide, exceeding the combined prevalence of the five most common noncommunicable diseases [1]. Oral dysbiosis connects systemic diseases, such as respiratory infections and cardiovascular diseases, to oral diseases, such as dental caries, periodontitis, and oral cancers [2,3]. Dental caries, impacting over 50% of the global population, particularly children, is highly preventable yet remains undertreated due to limited access to care, high costs, and low prioritization in healthcare systems. The rise in antimicrobial resistance (AMR) further complicates treatment, with oral antibiotic prescriptions accounting for 3–11% of total usage and often misused prophylactically or therapeutically for minor conditions [4,5]. To escape and survive in the host environment, biofilm disrupts the gene expression, thus enhancing the resistance to antibiotics [6].

Microorganisms in the marine environment are constantly evolving to adapt to their surroundings, making it a promising reservoir for potential novel drugs [7]. About 3.67 × 10^30^ microorganisms have been studied for their potential in the marine environment [8]. Marine microorganisms are the origins of many novel antimicrobial molecules [8], with over 50% of FDA-approved drugs derived from microbial natural products. Biosurfactants are surface-active compounds from microbes, which are biodegradable, stable under extreme conditions, and environmentally safe [9,10]. These molecules solubilize substances such as hydrocarbons, lipids, and oils and function as antibiotics by utilizing carbon and nitrogen sources from the environment. Biosurfactants can maintain a partition between the interphases of fluids due to their distinct polar properties and hydrogen bonding, thus effectively preventing the adhesion of microorganisms [11,12]. Biosurfactants offer a promising solution to these challenges due to their wide range of antimicrobial activity and low critical micelle concentration (CMC), enabling effective antimicrobial action at minimal doses.

*Bacillus* species, widely studied for their non-pathogenic nature, are important producers of biosurfactants. These species are particularly promising due to their antimicrobial, anticancer, and immunomodulatory properties, as well as their capacity to increase drug bioavailability and act as drug delivery vehicles [10,13,14]. *Bacillus* species produce diverse secondary metabolites with antimicrobial and antibiofilm properties, including lipopeptides and bacteriocins that disrupt bacterial cell membranes and quorum-sensing inhibitors that prevent adhesion and biofilm development. Crude lipopeptide extracts from *Bacillus subtilis* can reduce microbial adhesion up to 69% without host cytotoxicity [15], emphasizing their potential in oral pathogen control. Biofilm formation by *Bacillus* strains may influence biosurfactant production, reflecting ecological adaptability. Surfactin promotes early pellicle biofilm formation in *Bacillus subtilis,* demonstrating a functional connection between biosurfactants and biofilm initiation [16]. Pathogens such as *Streptococcus mutans*, *Streptococcus anginosus*, *Staphylococcus aureus*, and *Enterococcus faecalis* play key roles in these infections, exhibiting strong biofilm-forming capacity, which confers tolerance to antimicrobial agents and host responses [17,18]. Managing oral health is challenging due to the structural complexity of polymicrobial biofilms, particularly dental caries and periodontitis which provide a resilient physical barrier against both mechanical debridement and conventional antibiotics. Limitations in drug penetration often render conventional treatments ineffective. Mechanical removal often fails due to the resilient nature of biofilms, and long-term use of chemicals such as chlorhexidine associated with adverse side effects, including tooth staining, taste alteration, low pH, salivation, plasma nitrate concentration, increased systolic blood pressure and oral microbiome dysbiosis [19,20]. Consequently, there is an urgent need for novel biofilm targeted therapeutic strategies.

Marine ecotones, characterized by diverse environmental conditions such as fluctuations in salinity, pH, nutrient availability may serve as a unique hotspot for metabolic diversity. *Bacillus* species from these niches, evolve to produce structurally novel and robust biosurfactants with enhanced stability and antimicrobial properties, offering a promising, targeted platform for combating resilient oral pathogens [21]. Despite the vast potential of biosurfactants, their efficacy against oral pathogens remains underexplored. This study addresses the gap by exploring the antibacterial and antibiofilm potential of biosurfactants from marine *Bacillus* strains against oral pathogens. Additionally, this study supports the Sustainable Development Goals (SDGs) by advancing sustainable microbial-derived bioactive compounds as potential therapeutics aligning with SDG 3 (Good Health and Well-Being) and by promoting the use of eco-friendly biosurfactants through sustainable marine bioprospecting, thereby reducing environmental and aquatic toxicity in support of SDG 14 (Life Below Water) [22].

## 2. Materials and Methods

### 2.1. Chemicals

All chemicals and solvents used in this study were purchased from SRL Chemical Private Ltd., Chennai, India and culture media were procured from Himedia Laboratories Pvt Ltd. (Mumbai, India). The Blood agar plates were purchased from Biomerieux (Chennai, India), and the molecular reagents were purchased from Sigma Aldrich Chemicals Private Ltd. (Bangalore, India).

### 2.2. Isolation and Identification of Bacillus Strains

A total of seven marine samples (water and sediments) were collected, including six from four different locations along the Andaman and Nicobar Islands coastlines in the Indian Ocean and one from the Threspuram coast in Tuticorin, Tamil Nadu, situated along the Coromandel Coast of the Bay of Bengal, India. The samples were collected in a sterile plastic container, transported on ice, and stored at 4 °C. For processing, one gram of the sample was suspended in normal saline (1:9) and subjected to a heat-shock treatment by boiling at 100 °C for two minutes. This temperature and duration were specifically chosen to selectively isolate the *Bacillus* community while preventing the growth of heat-sensitive non-spore forming marine bacteria. Following the samples were serially diluted, and plated on Luria Bertani Miller (LB) agar medium (Himedia, Mumbai, India) was prepared by the pour-plate method and incubated overnight at 37 °C.

The preliminary identification of bacterial isolates was conducted by Gram staining and motility. Based on the macroscopic and microscopic morphological investigations, the bacterial isolates were stored in 25% glycerol at −20 °C until further usage. The genomic DNA was extracted using the hot alkaline-ethanol lysis method [23]. Polymerase Chain Reaction (PCR) (Himedia, Mumbai, India) amplification targeting the 16S rRNA gene was performed using universal forward primer 8F (5′-AGAGTTTGATCCTGGCTCAG-3′) and reverse primer 1541R (5′-AAGGAGGTGATCCAGCCGCA-3′) (Sigma Aldrich Chemicals Pvt Ltd.). *Bacillus subtilis* MTCC 2757 was used as a positive control. The detection of amplicon size (~1500 bp) was determined by 0.8% agarose gel electrophoresis; subsequently, the strains were confirmed by 16S rRNA gene sequencing (Applied Biosystems 3730xl DNA analyzer (Waltham, MA, USA)) using a one-way approach with the 8F forward primer. The raw sequence data were trimmed to remove low-quality bases, resulting in high-quality sequences of approximately 800 bp. The results were analyzed by the National Center for Biotechnology Information (NCBI) Nucleotide Basic Local Alignment Search Tool (nBLAST, https://blast.ncbi.nlm.nih.gov/Blast.cgi?PROGRAM=blastn&BLAST_SPEC=GeoBlast&PAGE_TYPE=BlastSearch, accessed on 20 November 2025). A phylogenetic tree was constructed to identify closely related species by the Maximum-Likelihood method using the Molecular Evolutionary Genetic Analysis (MEGA 12 version 12.0.9).

### 2.3. Screening of Biosurfactant-Producing Bacillus Strains

Biosurfactant producing ability of *Bacillus* strains was evaluated using qualitative (drop collapse assays, hemolytic activity, and bacterial adhesion to hydrocarbons (BATH)) and quantitative (oil displacement and emulsion index E24, and biofilm formation) assays.

#### 2.3.1. Hemolytic Activity

Overnight bacterial cultures were streaked onto Columbia blood agar with 5% sheep blood (Biomerieux, Chennai, India) and incubated overnight at 37 °C. The formation of a clear zone around the colonies indicated the biosurfactant production [24].

#### 2.3.2. Modified Drop Collapse Assay (MDC)

A drop of engine oil was placed on a glass slide, overlaid with cell-free supernatant (CFS). Collapse of the oil droplet indicates the presence of biosurfactants [24].

#### 2.3.3. Oil Displacement Assay

The distilled water was filled in a Petri plate, overlaid with 10 μL of crude oil. Then, 10 μL of the CFS was carefully dispensed at the center of the crude oil. After 30 s, the diameter of the clearance zone was measured, which is directly proportional to the surfactant concentration [24].

#### 2.3.4. Bacterial Adhesion to Hydrocarbons (BATH) Assay

Bacterial cell adherence to hydrocarbons (hydrophobicity) was assessed as described by Rosenberg et al. The bacterial pellet was rinsed with phosphate-buffered saline (PBS) twice and resuspended in PBS. The optical density (OD) was adjusted to 0.5 at 600 nm. Then 100 μL of crude oil was mixed with 2 mL of the cell suspension in a test tube and vortexed at maximum speed for 3 min. The mixture was then allowed to settle for one hour for separation, and later the OD of the aqueous phase was measured at 600 nm [25]. The percentage of bacterial cell adherence was calculated using the formula:

$$\mathrm{Percentage}\;\mathrm{of}\;\mathrm{bacterial}\;\mathrm{cell}\;\mathrm{adhesion}\;=\;1\;-\;\frac{OD\;of\;aqueous\;solution\left(treated\right)}{OD\;original\left(non-treated\right)}\times 100$$

#### 2.3.5. Emulsification Index (E_24_)

An equal volume of CFS and kerosene oil (2 mL each) was added to a test tube, vortexed at 3000 rpm for 2 min, and left undisturbed in the dark. After 24 h, emulsion formation was measured and calculated by the formula [7].

$$\mathrm{Emulsion}\;\mathrm{Index}\;(E_{24})=\frac{Height\;of\;emulsion\;layer}{Height\;of\;total\;liquid}\times 100$$

#### 2.3.6. Biofilm Formation Assay

The biofilm-forming capacity of 13 *Bacillus* strains was analyzed using a 96-well microtiter plate assay. Overnight cultures in tryptic soy broth (TSB) were adjusted to a 0.5 McFarland standard. A 10 μL of each *Bacillus* strain was inoculated into 190 μL of TSB in the flat-bottom microtiter plates and incubated at 37 °C for 4 days. The planktonic cells were removed by rinsing with PBS twice. The attached biofilms were stained with 200 μL of 0.1% crystal violet for 15 min at room temperature and rinsed again with the same buffer. About 150 μL of 33% acetic acid was added to solubilize the bound crystal violet, and absorbance (OD) was determined at 590 nm. The biofilm formed was calculated by the formula:

Biofilm formation = OD590 of the treated sample − OD590 of the untreated control (broth)

### 2.4. Antibacterial and Antibiofilm Activity of Bacillus Strains Against Oral Pathogens

#### 2.4.1. Preparation of Crude Extract of *Bacillus* Strains

The crude extract (CE) was prepared as described by Das et al. [26] with some modifications. Approximately 50 mL of an overnight bacterial culture was inoculated into 450 mL of ISP-1 medium and incubated in a shaker at 30 °C and 150 rpm for 7 days. The fermented culture was centrifuged at 8000 rpm for 10 min to remove cell debris. The resulting CFS was adjusted to pH 2.0 using 3 M HCl. The organic phase was extracted by adding ethyl acetate (1:1 ratio) to the CFS. The CE was subsequently concentrated and dried for further analysis.

#### 2.4.2. Source of Oral Pathogens

Oral pathogens, such as *Streptococcus mutans*, *Streptococcus anginosus*, *Staphylococcus aureus*, and *Enterococcus faecalis*, obtained from MTCC were used for the study. *Streptococcus mutans* (MTCC-497T) and *Streptococcus anginosus* (MTCC-1929) were cultured in the Brain Heart Infusion (BHI) medium, while *Staphylococcus aureus* (MTCC-96) and *Enterococcus faecalis* (MTCC-439) were grown in nutrient broth.

#### 2.4.3. Antibacterial Activity of Crude Extracts

The antibacterial activity of the CE was tested against oral pathogens by the agar well diffusion method, and the diameter of the clear zone was measured in millimeters [27]. *Staphylococcus aureus* and *Enterococcus faecalis* were cultured on Mueller-Hinton agar (MHA), while *Streptococcus mutans* and *Streptococcus anginosus* were grown on Columbia blood agar (Biomerieux). Overnight cultures were optimized to 0.5 McFarland standard (1.5 × 10^8^ CFU/mL) and evenly streaked on the respective agar plates. Wells of 6 mm diameter were created in each plate, into which 25 µL (0.05 g/mL) of crude was added. The results were observed after 24 h of incubation at 37 °C, and the size of the clear zone was measured. To validate the antimicrobial activity, standard antibiotics were used as positive controls. For *Enterococcus faecalis* and *Staphylococcus aureus,* Vancomycin (4 μg/mL) was used, while Penicillin (0.6 μg/mL) was used for *Streptococcus mutans* and *Streptococcus anginosus*. All antibiotics were obtained from HiMedia Pvt Ltd., Mumbai, India. The solvent (ethyl acetate) was used as the negative control.

#### 2.4.4. Antibiofilm Activity

The antibiofilm activity of CE from *Bacillus* strains with superior antibacterial activity was evaluated against the four oral pathogens using a 96-well microtiter plate assay [28]. Overnight cultures were optimized to a 0.5 McFarland standard. 100 μL of prepared culture was dispensed into flat-bottom polystyrene wells, followed by 100 μL of CE was added to each well at different concentrations, such as 125, 250, and 500 μg/mL. The microtiter plates were kept at 37 °C for 24–48 h to assess biofilm inhibition. The planktonic cells were rinsed thrice with PBS, and plates were air-dried for 30 min. The attached biofilms were stained with 150 μL of 0.1% crystal violet for 15 min at room temperature and later rinsed with PBS thrice. Then the same amount of 33% acetic acid was used to solubilize the biofilms, and the absorbance was recorded at 590 nm. The resulting OD values were normalized by adjusting the negative control as 0% and the untreated culture control as 100%. The antibiofilm activity was calculated by the formula:

Percentage of Biofilm Inhibition=[(OD of untreated control−OD of treated sample)]×100

### 2.5. Statistical Analysis

All assays were performed in duplicate, and the result values were recorded as mean ± standard error (SE). Statistical significance of differences among groups was evaluated by the ANOVA method, with a *p*-value threshold of <0.05 indicating significance. All statistical analysis and graphical representations were generated using Jamovi software 2.6.44.

## 3. Results and Discussion

### 3.1. Isolation and Identification of Bacillus Species

**Figure 1:**
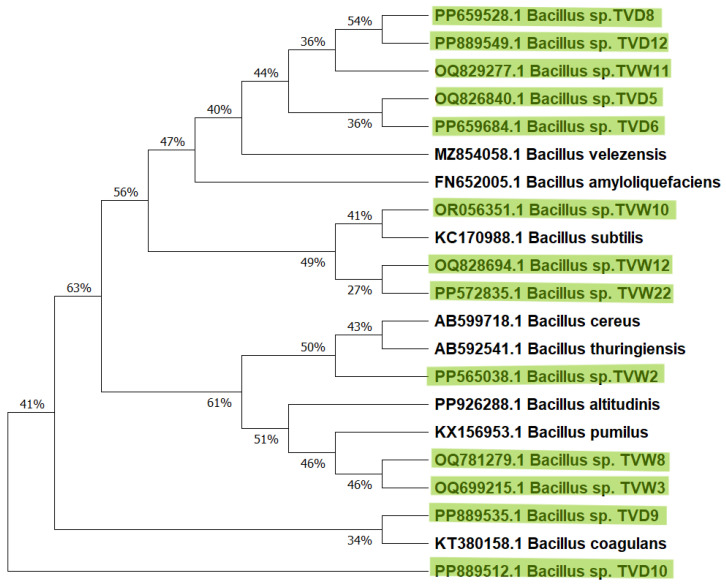
Phylogenetic tree depicting the evolutionary relationships of *Bacillus* strains, constructed using the Maximum likelihood method (MEGA 12). The tree with the highest log likelihood (−6862) is represented. *Bacillus* strains isolated in the present study are highlighted in green.

The eastern coastal regions of South India and the Andaman and Nicobar Islands, located near the Gulf of Mannar Marine National Park in the Bay of Bengal, are renowned for their exceptional biodiversity, hosting one of the world’s most diverse marine ecosystems. The ecological richness makes these areas ideal for exploring microbial diversity and novel metabolites. In this study, we selected these biodiverse coastal sites to isolate *Bacillus* species, assess their biosurfactant-producing ability, and evaluate antibacterial and antibiofilm potential against oral pathogens. Among seven marine samples, comprising four water and three sediment sources, 81 bacterial isolates were obtained. Morphologically, 13 bacterial isolates were identified as Gram-positive, rod-shaped, motile bacilli. Genomic DNA from these strains was extracted, and the 16S rRNA gene (~1500 bp) amplification was completed by PCR. Sanger sequencing and analysis using the NCBI nucleotide BLAST (https://blast.ncbi.nlm.nih.gov/Blast.cgi?PROGRAM=blastn&BLAST_SPEC=GeoBlast&PAGE_TYPE=BlastSearch, accessed on 20 November 2025) confirmed 98–100% similarity to the genus *Bacillus,* with sequences deposited in NCBI-GenBank [29] (Figure 1). This conservative approach was adopted because of the 800 bp sequence obtained from one-way sequencing, which was sufficient for genus-level identification, but does not provide enough phylogenetic depth to distinguish between closely related species within the *Bacillus* genus. To ensure taxonomic accuracy and avoid potential misidentification caused by ambiguous BLAST hits, these isolates were maintained as *Bacillus* sp. In accordance with previous studies, within the *Bacillus* species particularly *B. subtilis* and *B. cereus* complexes, the 16S rRNA gene exhibits >99.5% homology across its entire length. Therefore, with full length sequences (1500 bp), this gene is often insufficient to definitively discriminate between closely related without additional multi-locus sequence markers [30]. Phylogenetic analysis, conducted using the Maximum-Likelihood method by MEGA version 12, revealed that 80% of these isolates belong to the *Bacillus subtilis* group. The use of a Maximum Likelihood Phylogenetic tree (Figure 1) alongside the 800 bp sequence provides a scientific foundation for clade-level identification. This method ensures scientific accuracy and reliable basis for the functional interpretation of the isolate’s postbiotic potential. Also, the above findings were supported by previous studies in extreme environments, characterized by fluctuating altitudes, pH, temperature, light, and oxygen levels, which highlighted the critical role of *Bacillus* species in marine nutrient cycling. The current study in the Indian Ocean, Southern Ocean, and Bay of Bengal highlights their ecological significance in maintaining marine nutrient dynamics [31,32]. In the previous study, *Bacillus* species isolated from marine environments and oil reservoirs demonstrated that they produce non-hemolytic lipopeptide biosurfactants and F7 biosurfactants, effective against multidrug-resistant (MDR) pathogens, *Streptococcus mutans*, *Enterococcus faecalis*, and *Candida albicans*. Additionally, endophytic *Bacillus* strains yield antimicrobial peptides that inhibit biofilm formation, disrupt established biofilms, and suppress methicillin-resistant *Staphylococcus aureus* (MRSA) growth [26,33,34,35].

### 3.2. Screening of Biosurfactant-Producing Bacillus Species

#### 3.2.1. Hemolytic Assay

Thirteen *Bacillus* strains displayed varying biosurfactant production capacities. Beta-hemolytic strains are widely recognized for their superior biosurfactant synthesis compared to alpha- and gamma-hemolytic strains. Among the isolates, eight exhibited beta hemolysis, four showed alpha hemolysis, and one displayed gamma hemolysis, indicating a predominance of biosurfactant producers within the beta hemolytic group. Alpha and gamma hemolytic strains showed better oil displacement efficiency. In addition, *Bacillus* strain TVW2 is beta-hemolytic and demonstrates poor oil displacement compared to other isolated strains (Table 1).

#### 3.2.2. Drop Collapse Assay

Five *Bacillus* strains tested positive in the drop collapse assay (Table 1), with all except one beta hemolytic strain showing activity. In contrast, alpha and gamma hemolytic strains were consistently negative, suggesting that beta hemolysis strongly correlates with reduction in surface tension and collapse of oil droplets, a hallmark of biosurfactants. The drop collapse assay was sensitive and specific, detecting low biosurfactant concentrations. However, biosurfactant production was not exclusive to beta-hemolytic strains, as some non-beta-hemolytic strains may produce biosurfactants, while certain beta-hemolytic strains may not produce biosurfactants [36]. These results align with Satpute et al. [37], observing inconsistencies between hemolytic and drop collapse assays, suggesting hemolysis as an unreliable indicator of biosurfactant production. The drop-collapse assay alone was inconsistent for identifying biosurfactant-producing strains, necessitating multiple assays for accurate screening.

#### 3.2.3. Oil Displacement Assay

Oil displacement zones varied widely among the 13 marine *Bacillus* strains, ranging from 5.5 mm (*Bacillus* strain TVW22) to 19.5 mm (*Bacillus* strain TVW12). *Bacillus* strains TVW12 (19.5 mm), TVD8 (16.5 mm), and TVD6 (15 mm) exhibited clearance zones of ≥15 mm in the oil displacement assay. In contrast, three others (*Bacillus* strains TVW3, TVW8, and TVW10) were ≥10 mm, and the remaining six yielded zones ≤10 mm (Table 1), indicated as strong, moderate, and weak activity strains, respectively. These results are consistent with previous reports, such as *Bacillus* strain WD22 and *Bacillus cereus* BCS0 demonstrated high biosurfactant production, reinforcing the potential of marine-derived *Bacillus* biosurfactants [38,39]. Beta-hemolytic strains generally outperformed others, though two alpha-hemolytic strains also showed distinguished zones, indicating some biosurfactant production capacity across hemolytic types. Clearance zone size correlates directly with biosurfactant concentration, reflecting strain-specific production levels. Interestingly, *Bacillus* strain TVW22 (beta-hemolytic) was negative in the drop collapse assay and showed minimal oil displacement, while some strains exhibited contrary results. This lack of correlation between the two assays aligns with findings by Sun et al. [40]. Biosurfactants may not induce hemolysis when they are unable to penetrate agar, and low concentrations may not trigger oil droplet collapse in the drop collapse assay, yet still produce a clearance zone in the oil displacement assay. Consequently, the oil displacement assay is a reliable method for identifying biosurfactant-producing strains [36]. Although differences in oil displacement were not statistically significant (*p* = 0.3), the larger zones observed in TVW12, TVD8, and TVD6 highlight their promising biosurfactant potential. This variability may reflect differences in biosurfactant production pathways, meriting further investigation with larger sample sizes to elucidate underlying mechanisms.

#### 3.2.4. Bacterial Adherence to Hydrocarbons (BATH)

**Figure 2:**
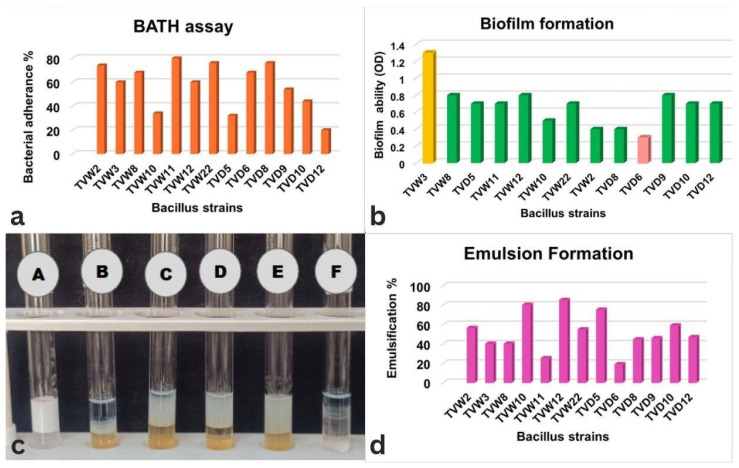
(**a**) Bacterial adherence to hydrocarbons (BATH assay) using cell-free supernatants (CFS) of *Bacillus* strains in percentage; (**b**) Biofilm formation of *Bacillus* strains: biofilm formation ability (OD in nm) ≥1.5 indicates strong (yellow), 1.0–1.5 indicates moderate (green), and <1.0 indicates weak (light pink); (**c**) Emulsion formation capacity (E_24_) in percentage; (**d**) Emulsion formation capacity (E_24_) using CFS of *Bacillus* species with kerosene oil. A. sodium dodecyl sulfate (SDS 10%) (positive control), B. *Bacillus* strain TVD6, C. *Bacillus* strain TVD8, D. *Bacillus* strain TVD10, E. *Bacillus* strain TVD5, and F. negative control (ddH_2_O).

Nine of the 13 marine *Bacillus* isolates exhibited hydrophobicity exceeding 50%, suggesting robust biosurfactant production [41]. *Bacillus* sp. TVW11 exhibited the highest cell surface hydrophobicity at approximately 77% (Figure 2a), consistent with prior studies that link biosurfactant activity to enhanced adhesion to hydrophobic surfaces [42]. Biosurfactants, characterized by low surface energy, facilitate microbial adherence to hydrocarbons, a hallmark of their activity [43]. A positive correlation exists between biosurfactant production and cell hydrophobicity, with strains producing higher biosurfactant quantities typically showing greater adhesion [44]. For instance, *Bacillus tequilensis* MK 729017 achieved maximum hydrophobicity of 52 ± 0.5% at 72 h, coinciding with peak biosurfactant concentration and correlating with efficient hydrocarbon degradation [45]. Meanwhile, *Bacillus* strain TVW11’s superior hydrophobicity suggests it is a highly promising biosurfactant producer, potentially outperforming previously studied strains like *B. tequilensis* MK 729017. High hydrophobicity typically correlates with efficient hydrocarbon degradation and strong interaction with oil phases, complementing E_24_ results.

#### 3.2.5. Emulsion Index (E_24_)

The E_24_ index indirectly measures biosurfactant concentration, with higher values signaling greater production, reflecting surfactant concentration and stability. It is reported that more than 30% of the emulsion index suggested good biosurfactant production [46]. *Bacillus* strain TVD5 demonstrates good emulsification potential (Figure 2b,c), similar to *Bacillus tequilensis* MK 729017, which recorded an E24 of 66 ± 2% with crude oil and reduced surface tension to 30 ± 2 mN/m due to a lipopeptide surfactin biosurfactant [45]. The findings also demonstrated that few strains exceeded more than 50% emulsion formation after 24 h, suggesting that they produce stable and substantial biosurfactants, possibly of high molecular weight [47]. Similarly, a study evaluating six *Bacillus* species with glucose and xylose as carbon sources reported E24 values above 50% for all strains [48]. *Bacillus* strains TVW11, TVD9, and TVD12 exhibit low emulsification values, suggesting either less stable compounds or reduced extracellular emulsification production.

The high proportion of beta-hemolytic strains among these marine isolates may reflect an adaptive response to extreme environmental conditions, with biosurfactant production enhancing survival, as supported by previous studies [43,49,50]. However, the association between beta-hemolysis and biosurfactant production requires further investigation. These findings indicate that the hemolytic assay alone is an unreliable indicator of biosurfactant production, as it may yield false positives or negatives. To accurately assess biosurfactant capabilities, complementary assays such as oil displacement, drop collapse, BATH, and emulsification index are recommended [37,51].

#### 3.2.6. Biofilm Formation

The biofilm-forming capacity of 13 marine *Bacillus* strains was analyzed using a microtiter plate (96-well) assay. *Bacillus* strain TVW3 exhibited moderate biofilm formation, the highest among the strains, while the remaining 12 strains were grouped as weak biofilm producers, as represented in Figure 2d. This variability suggests a complex interplay between biofilm formation and biosurfactant production in marine *Bacillus* isolates. Surfactin exhibits a dose-dependent dual role: at low concentrations, it promotes biofilm initiation by signaling molecules and induces potassium leakage to activate *spo0A*, a regulator of sporulation and biofilm formation [52,53]. At higher concentrations, surfactin reduces surface tension, facilitating cell motility and dispersal from mature biofilms, thus modulating hydrophobic interactions to form structured biofilms [16]. Biosurfactant genes and biofilm matrix genes are co-regulated by *spo0A*, *comA*, and *degU* [54], indicating a shared regulatory network. This dose-dependent relationship suggests that *Bacillus* strain TVW3 could be leveraged for antibiofilm strategies, though extensive studies with larger sample sizes are mandatory to elucidate these underlying mechanisms [55].

### 3.3. Antibacterial and Antibiofilm Activity of Bacillus Strains Against Oral Pathogens

#### 3.3.1. Antibacterial Activity of Crude Extracts

**Figure 3:**
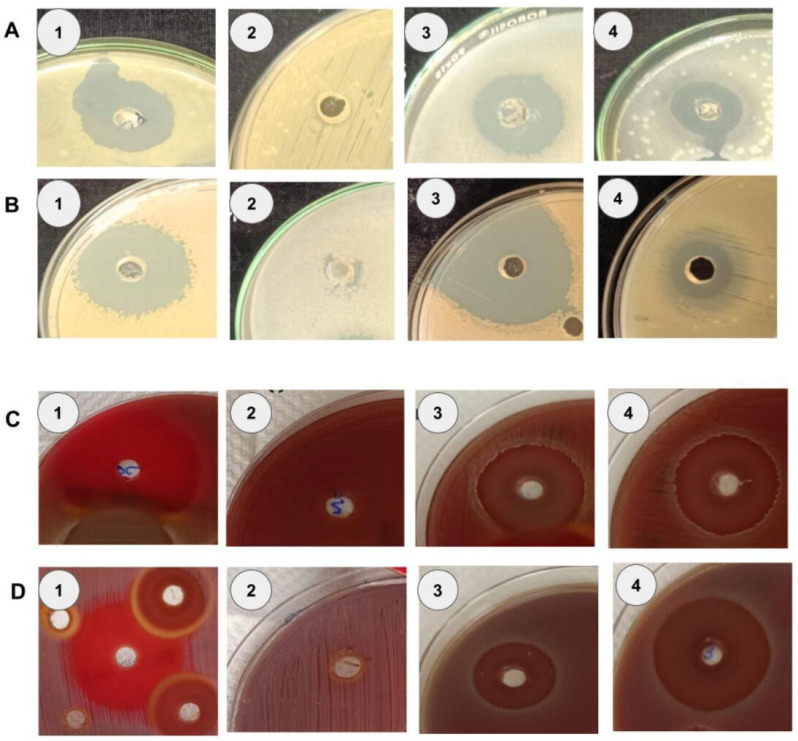
Antibacterial activity of crude extracts of marine *Bacillus* strains TVD9 and TVD10 against four oral pathogens: Zone of clearance—(**A**). *Enterococcus faecalis*: A1 positive control (vancomycin (4 μg/mL)—25 μL), A2 negative control (ethyl acetate pure—25 μL), A3 *Bacillus* strain TVD9 and A4 *Bacillus* strain TVD10; (**B**) *Staphylococcus aureus*: B1 positive control (vancomycin (4 μg/mL)—25 μL), B2 negative control (ethyl acetate pure—25 μL), B3 *Bacillus* strain TVD9 and B4 *Bacillus* strain TVD10; (**C**) *Streptococcus mutans*: C1 positive control (penicillin (0.6 μg/mL)—25 μL), C2 negative control (ethyl acetate pure—25 μL), C3 *Bacillus* strain TVD9 and C4 *Bacillus* strain TVD10; and (**D**) *Streptococcus anginosus*: D1 positive control (penicillin (0.6 μg/mL)—25 μL), D2 negative control (ethyl acetate pure—25 μL), D3 *Bacillus* strain TVD9 and D4 *Bacillus* strain TVD10.

The antibacterial efficacy of CE from 13 marine *Bacillus* strains was evaluated against oral pathogens such as *Streptococcus mutans*, *Streptococcus anginosus*, *Staphylococcus aureus*, and *Enterococcus faecalis*, using the agar-well diffusion method. The crude extracts from six *Bacillus* strains, such as TVW10, TVW22, TVD8, TVD9, TVD10, and TVD12 displayed significant antibacterial activity against all four oral pathogens, with TVD9 (beta-hemolytic) and TVD10 (alpha-hemolytic) extracts showing no hemolytic activity on blood agar, indicating their potential as non-hemolytic antibacterial agents. *Bacillus* TVD9 beta hemolytic strain tested positive for drop collapse but showed moderate oil clearance, while *Bacillus* TVD10 alpha hemolytic strain was negative for drop collapse and also had limited oil clearance. The results align with Das et al. [26], who identified a non-hemolytic lipopeptide biosurfactant from marine *Bacillus circulans* with similar antimicrobial properties. Overall, these extracts effectively inhibited pathogen growth, with eight strains producing inhibition zones ≥ 20 mm against *S. mutans*, six yielding zones ≥ 18 mm against *S. anginosus*, two achieving zones ≥ 15 mm against *S. aureus*, and two exhibiting zones ≥ 20 mm against *E. faecalis* (Table 2, Figure 3). While most strains exhibited broad spectrum efficacy, *Bacillus* sp. TVW2, lacked activity against *S. aureus* and *E. faecalis*, and *Bacillus* sp. TVW3, was ineffective against *E. faecalis*. Conversely, *Bacillus* sp. TVD10 demonstrated significant potential with zones of 32 ± 1.4 mm against *S. mutans* and 31.5 ± 2.1 mm against *S. anginosus*, which are comparable to the penicillin positive control (35.5 ± 0.7 mm and 32 ± 1.4 mm, respectively). *Bacillus* strains TVW22 and TVD9 against *S. aureus* (MTCC 96) produced zones of 15 ± 0 mm and 16 ± 1.4 mm, respectively, while four strains exceeded 16 mm against *E. faecalis*, with *Bacillus* strain TVD10 reaching 25 ± 1.4 mm. These results compare favorably to Thando et al. [56], observing that *Bacillus amyloliquefaciens* ST34 extract had zones of 15.3 ± 0.5 mm against *S. aureus* (MRSA Xen 30) and 18.7 ± 0.9 mm against *E. faecalis*, versus 17.8 ± 0.8 mm for pure surfactin against *S. aureus* (ATCC). Given that some biosurfactants lack hemolytic activity and that clearing zones may be facilitated by lytic enzymes or other substances. These disparities imply that biosurfactants may not be the only source of antibacterial activity. The antimicrobial activity observed in the crude extracts classified as postbiotics, cell-free, non-viable metabolites like lipopeptides and antimicrobial peptides confer biological benefits. These findings highlight the diverse therapeutic potential of these secreted postbiotic components against the oral pathogens. This requires further purification and analysis of the crude extracts to identify the responsible compound for the observed effects.

Two-way ANOVA was conducted to analyze the antimicrobial activity of the *Bacillus* strains against the oral pathogens. The analysis revealed *Bacillus* strains (F_(13,56)_ = 11.23, *p* < 0.001 and the target pathogens (F_(3,56)_ = 20.77, *p* < 0.001) exerted a significant influence on the zones of inhibition. However, no significant interaction was observed between strain and pathogen (F_(39,56)_ = 0.765, *p* = 0.809). This result indicates that the inhibitory potential of each *Bacillus* strain remained relatively consistent regardless of the oral pathogen tested, highlighting a broad-spectrum and stable antimicrobial potential across the strains.

#### 3.3.2. Antibiofilm Activity of Crude Extracts

**Figure 4:**
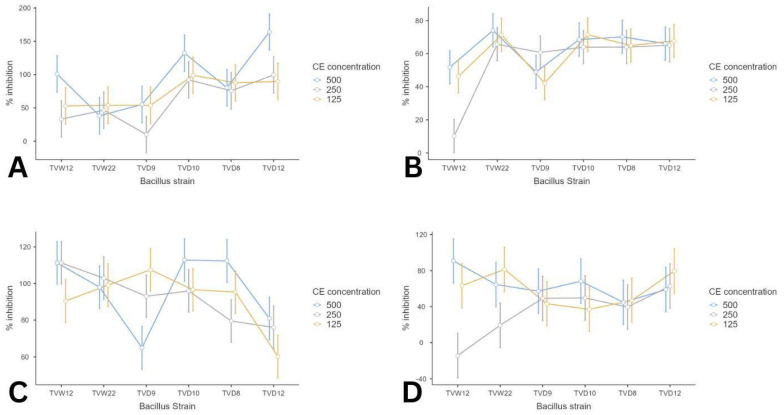
Antibiofilm activity of selected *Bacillus* strains at different concentrations: 500 μg/mL, 250 μg/mL, and 125 μg/mL against oral pathogens. (**A**) *E. faecalis*; (**B**) *S. anginosus*; (**C**) *S. aureus*; (**D**) *S. mutans*.

Six *Bacillus* strains, such as TVW12, TVW22, TVD8, TVD9, TVD10 and TVD12 demonstrated significant antibacterial activity against four oral pathogens and were analyzed for antibiofilm assay as depicted in Figure 4. The CE of *Bacillus* strain TVW12, and TVD12 shows maximum inhibition (~60%) against *Streptococcus mutans*. (Figure 4D) This highlights the good antibiofilm activity of CE in conditions where biofilm formation is less dense. *Bacillus* strain TVW22, TVD10, TVD8, and TVD9 exhibit good inhibition (~65%) against *Streptococcus anginosus*. (Figure 4B) *Streptococcus anginosus* exhibits strong biofilm formation mediated by AI-2 quorum sensing through the *LuxS* pathway [57]. This relatively high baseline biofilm density (0.81) may be attributed to the reduced inhibitory effect of crude extract. Six *Bacillus* strains demonstrated good inhibition > 60% against *Staphylococcus aureus*. (Figure 4C) Previous studies reported that *Bacillus cereus* biosurfactants demonstrated a significant antibiofilm effect (~80%) against *S. aureus* in combination with *Serratia nematodiphila* biosurfactants [58]. *Bacillus subtilis* 6D1 effectively disassembled the mature MRSA biofilm and interfered with quorum sensing, enhancing antibiotic sensitivity. In the previous study, the cell-free supernatants of marine-derived *Bacillus subtilis* MTUA2 and *Bacillus velezensis* MTUC2 significantly inhibited both early and established biofilms of various pathogens, including *S. aureus,* by bacteriocins such as subtilosin, subtilomycin, and plantazolicin [57]. In the present study, *Bacillus* strains TVD10, TVD8, TVD12 and TVW12 (Figure 4A) exhibited > 80% inhibition against *E. faecalis* at all different concentrations. Intrinsic resistance of *E. faecalis* to many antibiotics and robust biofilm-forming ability, it shows strong resilience in the root canal environment [59]. Our findings suggest that *Bacillus-*derived natural compounds exhibit significant antibiofilm efficacy against these oral pathogens. The above findings reveal that these postbiotic extracts also demonstrated significant antibiofilm potential by inhibiting initial attachment and disrupting established biofilms. Its high effectiveness against *S. mutans* and *S. aureus,* both weaker biofilm producers, underscores its potential use in early-stage or less robust biofilm contexts.

Three-way ANOVA was conducted to analyze the effects of *Bacillus* strains, oral pathogens, and concentration on inhibition assay. The result revealed significant interaction between the strains and the pathogens (F_(15,72)_ = 2.575, *p* = 0.004), confirming that the inhibitory efficacy is pathogen-specific. This indicates that the potential of each *Bacillus* strain depends significantly on the specific oral pathogen being targeted. However, no significant interaction was observed between strain and concentration (F_(10,72)_ = 1.061, *p* = 0.403) or in the three-way interaction (F_(30,72)_ = 0.758, *p* = 0.798), suggesting that while the strains are pathogen-specific, their relative performance remains consistent across the tested concentrations.

Curcumin, a well-studied natural compound, inhibits *S. mutans* biofilm by disrupting EPS synthesis and acid tolerance mechanisms, including affecting F-ATPase and glucan binding proteins [60,61]. In a clinical setting, plant extracts and essential oils exhibit inhibitory effects against oral pathogens, occasionally approaching the levels of chlorhexidine [62]. Particularly, cranberry extracts show dose-dependent biofilm reduction in *S. mutans* [57]. These findings support our results that the integrity of the *S. mutans* biofilm can be successfully disrupted by *Bacillus* species.

Surprisingly, some of the *Bacillus* strains displayed high antibiofilm activity occurring at lower (125 μg/mL) concentration with reduced efficiency at higher (250 μg/mL and 500 μg/mL) concentrations, suggesting a non-monotonic, window-dependent dose–response. This trend mirrors observations in previous studies with surface-active metabolites, demonstrating antimicrobial potency often peaks near the critical micelle concentration (CMC). Above the CMC, active monomers aggregate into micelles, reducing the availability to interact with microbial cells or disrupt extracellular polymeric substances (EPS) [63]. Moreover, in hermetic-dose-responses, low or intermediate doses inhibit biofilm formation more efficiently than higher doses, which was also documented for antimicrobial agents and biosurfactants. This biphasic behavior may result from higher concentrations triggering microbial stress responses or altering surface adhesion properties, which mitigate net inhibition [64,65]. With crude extracts, increasing concentration can also concentrate antagonistic constituents, enhance viscosity or lead to precipitation, each of which may reduce the antibiofilm potential [66]. Taken together, the antibiofilm activity of *Bacillus* strains is pathogen-specific, and the mechanisms might support a model where intermediate CE doses maximize bioactivity availability and disruption of biofilm integrity, while higher doses lead to self-assembly or physico-chemical changes that weaken overall efficiency.

The findings of this study have prominent clinical implications in the development of novel therapeutics in oral health. The antimicrobial activity observed is mediated by *Bacillus*-derived biosurfactants, particularly low molecular weight lipopeptides produced by ribosomal or nonribosomal pathways [67]. These lipopeptides intercalate into the bacterial plasma membrane, leading to increased permeability, the leakage of cytoplasmic components, resulting in cell lysis [68,69]. Furthermore, these postbiotic biosurfactants play a key role in clinical biofilm management, by disrupting the extra polymeric substances (EPS). This disruption not only facilitates antibiotic penetration but also interferes with quorum-sensing pathways, thereby attenuating pathogen virulence without necessarily inducing selective pressure for resistance [70]. Such studies are highly relevant in dentistry, as lipopeptide biosurfactants from *Bacillus subtilis* (ATCC 19659) demonstrated efficacy in preventing *S. aureus* and *Streptococcus sanguinis* biofilm formation on titanium dental implant surfaces [70]. *Bacillus*-derived postbiotics offer a selective advantage over synthetic surfactants such as sodium lauryl sulphate (SLS), and chlorhexidine which are highly associated with mucosal irritation, microbiome dysbiosis and systemic health concerns [19]. These findings support a paradigm shift in oral disease management towards the use of bioactive coatings for dental implants and orthodontic appliances. These applications are critical measures for preventing biofilm colonization by cariogenic pathogens implicated in peri-implantitis and other clinical complications [71].

## 4. Conclusions

The study highlights the marine environment as a valuable source of *Bacillus* species capable of producing biosurfactants with antibacterial and antibiofilm properties. It demonstrates that these species, isolated from diverse marine ecosystems beyond oil-contaminated sites, synthesize biosurfactants with significant efficacy. These strains possess a dual therapeutic profile characterized by consistent, broad-spectrum antimicrobial activity coupled with specialized, pathogen-specific antibiofilm efficacy. This unique combination suggests that these marine-derived postbiotics may serve as versatile agents for reducing oral microbial loads, while also providing a targeted platform for disrupting resilient, species-specific oral biofilms. These findings pave the way for future extensive experimental studies to further explore the clinical applications of marine biosurfactants to mitigate AMR challenges. Thus, this research supports the development of sustainable alternatives for the management of polymicrobial oral infections.

## Figures and Tables

**Table 1:** Summary of hemolytic activity, drop collapse assay, and oil displacement assay (n = 2) of marine *Bacillus* strains.

*Bacillus* Strains	Hemolytic Activity	Drop Collapse Assay	Oil Displacement Assay (ODA) Mean ± SE (mm)
*Bacillus* sp. TVW2	β	–	6 ± 4.73
*Bacillus* sp. TVW3	α	–	11 ± 1.4.73
*Bacillus* sp. TVW8	γ	–	7.5 ± 4.73
*Bacillus* sp. TVW10	α	–	7.5 ± 4.73
*Bacillus* sp. TVW11	α	–	14 ± 4.73
*Bacillus* sp. TVW12	β	+	16.5 ± 4.73
*Bacillus* sp. TVW22	β	–	5.5 ± 4.73
*Bacillus* sp. TVD5	β	+	8 ± 4.73
*Bacillus* sp. TVD6	β	+	13 ± 4.73
*Bacillus* sp. TVD8	β	+	16.5 ± 4.73
*Bacillus* sp. TVD9	β	+	9 ± 4.73
*Bacillus* sp. TVD10	α	–	4.5 ± 4.73
*Bacillus* sp. TVD12	β	+	7.5 ± 4.73

In drop collapse assy, + positive, – negative.

**Table 2:** Antibacterial activity of crude extracts of marine *Bacillus* strains against the oral pathogens.

*Bacillus* Strain	*S. mutans*	*S. anginosus*	*S. aureus*	*E. faecalis*
TVW2	11.5 ± 4.24	9 ± 4.24	0	0
TVW3	11.5 ± 4.24	8 ± 4.24	7 ± 4.24	6.5 ± 4.24
TVW8	20.5 ± 4.24	15.5 ± 4.24	7 ± 4.24	15.5 ± 4.24
TVW10	24.5 ± 4.24	20.5 ± 4.24	11 ± 4.24	17 ± 4.24
TVW11	22 ± 4.24	18 ± 4.24	10.5 ± 4.24	14 ± 4.24
TVW12	28 ± 4.24	21.5 ± 4.24	14 ± 4.24	19.5 ± 4.24
TVW22	29.5 ± 4.24	19 ± 4.24	15 ± 4.24	16 ± 4.24
TVD5	12 ± 4.24	7 ± 4.24	9 ± 4.24	9 ± 4.24
TVD6	13 ± 4.24	10.5 ± 4.24	6 ± 4.24	10.5 ± 4.24
TVD8	24.5 ± 4.24	18.5 ± 4.24	11 ± 4.24	19 ± 4.24
TVD9	27 ± 4.24	23.5 ± 4.24	16 ± 4.24	20 ± 4.24
TVD10	32 ± 4.24	31.5 ± 4.24	9 ± 4.24	25 ± 4.24
TVD12	31 ± 4.24	30.5 ± 4.24	20 ± 4.24	29 ± 4.24
PC	35.5 ± 4.24 *	18 ± 4.24 *	22 ± 4.24 **	26 ± 4.24 **

Notes: The diameter of the zone of inhibition in mm * Penicillin (0.6 μg/mL)—25 μL, ** Vancomycin (4 μg/mL)—25 μL (4 μg/mL)—25 μL, n = 2. Values are represented as Estimated Marginal Means ± Standard Error (SE) derived from the ANOVA model.

## Data Availability

The data presented in this study are openly available in NCBI at https://www.ncbi.nlm.nih.gov/ (accessed on 7 June 2024), reference numbers PP565038.1, PP659528.1, PP572835.1, OQ826840.1, OQ829277.1, OQ781279.1, OQ699215.1, PP659684.1, OQ828694.1, OR056351.1, PP889512.1, PP889535.1, PP889549.1.
